# Diagnostic Accuracy of Intestinal Parasitic Infections in Individuals Admitted to Medical Laboratories

**Published:** 2018-04

**Authors:** Fares BAHRAMI, Ali HAGHIGHI, Mohammad Bagher KHADEM-ERFAN, Ghasem ZAMINI

**Affiliations:** 1. Zoonoses Research Center, Kurdistan University of Medical Sciences, Sanandaj, Iran; 2. Dep. of Medical Parasitology and Mycology, School of Medicine, Shahid Beheshti University of Medical Sciences, Tehran, Iran; 3. Dep. of Parasitology and Mycology, Faculty of Medicine, Kurdistan University of Medical Sciences, Sanandaj, Iran; 4. Cellular and Molecular Research Center, Kurdistan University of Medical Sciences, Sanandaj, Iran

## Dear Editor-in-Chief

Intestinal parasitic infections (IPIs) are among the main health problems worldwide. IPIs can be caused by protozoan organisms or helminthes. The most common intestinal pathogenic parasites include *Giardia lamblia*, *Entamoeba histolytica/dispar*, *Cryptosporidium* spp., *Microsporidia* spp., *Cyclospora cayetanenensis*, *Ascaris lumbricoides*, *Ancylostoma duodenale*, *Blastocystis* sp. *Necator americanus*, *Hymenolepis nana*, *Taenia saginata*, and *Trichuris trichiura* (1, 2).

Misdiagnosis is a common medical laboratory error and can result in impact quality of life and unnecessary drugs use. Errors in medical laboratories do not always lead to vital threat, but the importance of misdiagnosis-related the social damages are great (3, 4).

Based on the unpublished data from laboratories of the Kurdistan province, misdiagnosis happen frequently in reports of IPIs, in the present study, we attempted to repeat, evaluate and compare the diagnoses results of IPIs from medical laboratories in Sanandaj, west of Iran. This investigation determined the diagnostic errors among medical laboratories for IPIs. This aim was accomplished by comparing the routine examinations results of the medical laboratories for diagnosis of IPIs by applying the same routine examinations and complementary methods as well.

This study was carried out according to the ethical guidelines of Shahid Beheshti University of Medical Sciences. Among available data on 694 individuals who admitted to 14 medical laboratories in Sanandaj City from July 2015 to November 2016.

The stool samples and recorded reports from laboratories were gathered randomly, then were transferred to the Parasitology Research Laboratory of Kurdistan University of Medical Sciences, Kurdistan, Iran and were examined again using routine stool examination (saline and Lugol wet mount) and also complementary methods (formalin ether concentration, acid-fast and tri-chrome staining) in accordance with standard protocols (5, 6) and CDC (7).

There were differences between our tests‘ results and medical laboratories‘ records; these differences were confirmed by applying complementary techniques. The misdiagnoses occurred in 95 (13.7%) subjects, including 73 (10.5%) and 22 (3.2%) as false-negative and false-positive respectively (Table 1). The most common mistakes as false negatives and false positives were *Blastocystis* sp. and *Entamoeba histolytica*/*dispar* respectively. The formalin ether concentration method was more sensitive for detection of amebiasis than direct smears. Many false-positive tests usually result from confusion of amebic trophozoites with macrophages ingested red cells and the misidentification of nonpathogenic protozoa for amebas (8).

**Table 1:** Frequency of false diagnoses of IPIs in 14 med. Labs in Sanandaj, western Iran

***Total cases (%)***	***False positive (%)***	***False negative (%)***	***Total false (%)***
694, (100)	22, (3.2)	73, (10.5)	95, (13.7)

Although these results emphasized the capability of formalin ether concentration technique and other complementary methods for the best detections of IPIs, but misdiagnoses may result in as Staffs’ errors, their poor skills, low quality of applied routine methods and the reporting of some controversial nonpathogenic parasites (like *Blastocystis* sp.) are unnecessary.

Low income, overwork and insufficient time, job dissatisfaction, confusion of parasites with parasite-like bodies or artifacts (such as macrophages and PMN cells) are among the others more common causes of misdiagnosis among the medical laboratory technicians.

In most laboratories in developing countries, the direct wet mount is the preferred stool parasitological detection technique because this method is simple, rapid, and inexpensive (9). Many of IPIs may be difficult or even impossible to detect with routine methods and require complementary methods. However, expertise in these methods is still essential to achieve ample sensitivity and avoid misdiagnosis (10).

This study showed that the accuracy of reported IPIs at the medical laboratories are low and can be enhanced by different ways such as: The employment of formol-ether concentration technique and if possible staining methods as a confirmatory test in routine laboratory examinations of stools or at least reexamining of three or more wet mount tests on separate days. Allocating sufficient time for investigation along with retraining of staffs will significantly reduce misdiagnosis of IPIs and its concomitant public health consequences.
